# Pre-ablation dual-energy CT-derived extracellular volume fraction predicts recurrence after radiofrequency ablation in non-paroxysmal atrial fibrillation

**DOI:** 10.1093/bjr/tqag149

**Published:** 2026-06-18

**Authors:** Shuangxiang Lin, Kaiqi Lv, Chengjia Liu, Shuyue Wang, Jiaxing Wu, Xinhong Wang, Jianzhong Sun

**Affiliations:** Department of Radiology, The Second Affiliated Hospital Zhejiang University School of Medicine, Hangzhou, Zhejiang 310000, China; Department of Cardiology, The Second Affiliated Hospital Zhejiang University School of Medicine, Hangzhou, Zhejiang 310000, China; Department of Radiology, The Second Affiliated Hospital Zhejiang University School of Medicine, Hangzhou, Zhejiang 310000, China; Department of Radiology, The Second Affiliated Hospital Zhejiang University School of Medicine, Hangzhou, Zhejiang 310000, China; DI CT, Siemens Healthineers, Shanghai 200120, China; Department of Radiology, The Second Affiliated Hospital Zhejiang University School of Medicine, Hangzhou, Zhejiang 310000, China; Department of Radiology, The Second Affiliated Hospital Zhejiang University School of Medicine, Hangzhou, Zhejiang 310000, China

**Keywords:** computed tomography angiography, atrial fibrillation, extracellular volume fraction, recurrence

## Abstract

**Objectives:**

To determine whether pre-ablation left ventricular extracellular volume fraction (ECV), derived from delayed-phase dual-energy cardiac computed tomography angiography (CTA), predicts atrial fibrillation (AF) recurrence after radiofrequency ablation in patients with non-paroxysmal AF.

**Methods:**

In this retrospective cohort, 399 patients with non-paroxysmal AF undergoing radiofrequency ablation were included. All patients underwent preprocedural cardiac CTA with delayed-phase imaging acquired 5 minutes after contrast administration to quantify left ventricular ECV. To identify independent predictors of AF recurrence, patients were stratified by an optimal ROC-derived cutoff and analyzed using univariate and multivariate Cox proportional hazards models. Model performance was assessed using the concordance index (C-index), net reclassification index (NRI), and integrated discrimination improvement (IDI).

**Results:**

During 10.1 months of follow-up (interquartile range, 8.1-13.6), 91 patients (22.8%) experienced AF recurrence after post-ablation. Recurrence was significantly associated with higher ECV values (37.6% ± 5.05% vs. 32.6% ± 2.05%; *P < .*01). The optimal ECV cutoff of 34.69% demonstrated an area under the curve (AUC) of 0.85 (95% CI: 0.79-0.90). Multivariate Cox regression identified high ECV (HR = 1.84; 95% CI: 1.24-2.33; *P < .*01) as independent predictors of AF recurrence. Additionally, integrating ECV with traditional clinical risk factors significantly enhanced predictive performance, achieving a C-index of 0.82 (95% CI: 0.74-0.86), an IDI of 0.66 (95% CI: 0.41-0.91), and an NRI of 0.55 (95% CI: 0.26-0.61).

**Conclusions:**

In non-paroxysmal AF patients, myocardial ECV derived from delayed-phase CT is an independent predictor of AF recurrence.

**Advances in knowledge:**

This study provides evidence that DECT-derived ECV, a surrogate marker of diffuse myocardial fibrosis, can stratify recurrence risk in non-paroxysmal AF patients.

## Introduction

Atrial fibrillation (AF) recurrence following catheter ablation remains a significant clinical challenge, with reported recurrence rates reaching up to 50% in some series.^1^^,^ ^2^ Non-paroxysmal AF, encompassing persistent and long-standing persistent forms, presents substantial management challenges.^3^^,^ ^4^ These patients typically exhibit more extensive myocardium remodeling, characterized by fibrosis and dilation, which contributes to a heightened risk of recurrence following rhythm control interventions such as radiofrequency ablation (RFA).^5^^,^ ^6^

Myocardial extracellular volume (ECV) quantification provides a noninvasive proxy for diffuse fibrosis by estimating the proportion of extracellular matrix within the myocardial tissue. While ECV has traditionally been quantified by T1 mapping on cardiac MRI, advances in imaging technology have enabled ECV measurement using cardiac computed tomography.^7^^,^ ^8^ CT-derived ECV uses paired pre- and post-contrast scans to estimate iodine distribution volume and has shown strong correlation with MRI-based ECV.^9^ Compared to MRI, CT imaging can be acquired more rapidly and is less restrictive for patients with metal implants, claustrophobia, or poor breath-holding ability. Specifically, delayed-phase cardiac CTA offers rapid, comprehensive, and cost-effective imaging, allowing simultaneous evaluation of coronary anatomy with submillimeter resolution.^10^^,^ ^11^ Notably, this high spatial resolution has recently made the quantification of atrial ECV and detailed regional atrial assessment technically feasible, largely overcoming the partial-volume limitations associated with MRI.^12^^,^ ^13^ While regional atrial mapping represents a promising emerging frontier for localizing fibrotic remodeling, established ventricular myocardial ECV serves as a robust and highly reproducible surrogate for the global profibrotic state and atrial myopathy.^14^^,^ ^15^

We hypothesized that elevated myocardial ECV would be associated with a greater burden of structural remodeling in AF patients and thus predict higher risk of AF recurrence after ablation. Therefore, this study aimed to determine whether CT-derived ECV could independently predict AF recurrence following RFA in patients with non-paroxysmal AF.

## Methods

### Study population

This retrospective study followed the Helsinki Declaration and was approved by the institutional review board (I2024450). The local Institutional Review Board approved this retrospective study and waived the need for written informed consent.

We enrolled 550 patients with non-paroxysmal AF who underwent pulmonary vein RFA between June 2022 and June 2024. Pre-ablation left atrial CTA was routinely performed to exclude left atrial appendage thrombus and assess atrial anatomy. Inclusion criteria were age 18 years or older, a diagnosis of persistent or long-standing persistent AF, and eligibility for contrast-enhanced cardiac CTA with delayed-phase imaging. Exclusion criteria included prior AF ablation, history of myocardial infarction, percutaneous coronary intervention (PCI), coronary artery bypass grafting (CABG), percutaneous balloon angioplasty (POBA), cardiac amyloidosis or other cardiomyopathies, contraindications to iodinated contrast media (severe renal impairment or contrast allergies), poor image quality due to respiratory motion artifacts, and inability to comply with follow-up protocols.

All participants underwent a comprehensive preprocedural evaluation, including detailed medical history, standard blood tests, and transthoracic echocardiography (TTE). TTE measurements, performed according to American Society of Echocardiography guidelines, included left ventricular ejection fraction (LVEF), and left atrial volume. Electrophysiologists performing the ablation procedures and subsequent clinical follow-up were blinded to the ECV measurements obtained from the preprocedural cardiac CT. The patient enrollment flow chart is presented in Figure 1.

**Figure 1:**
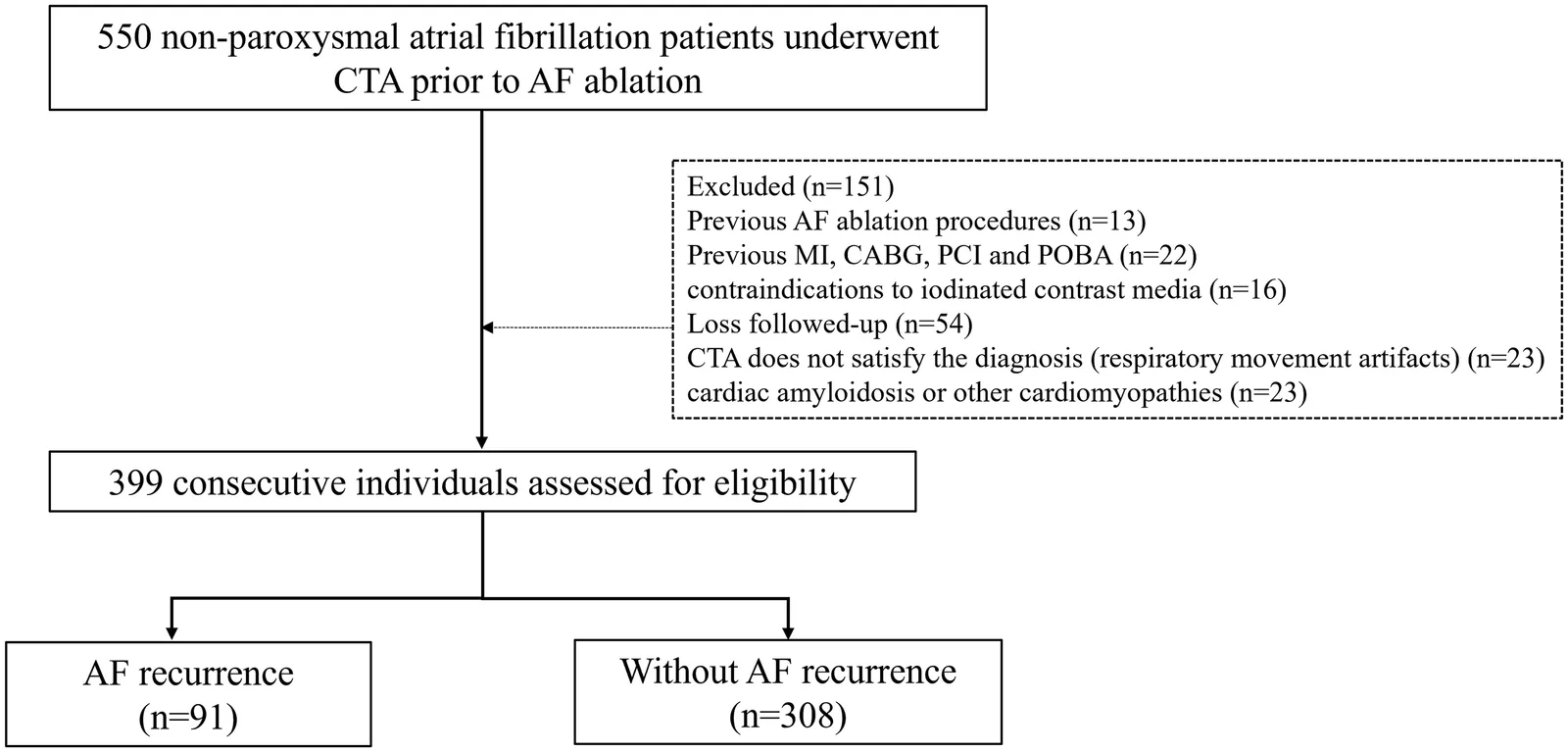
The flowchart of study population.

### CT acquisition protocol

All participants underwent prospective cardiac CTA using a third-generation dual-source CT scanner (SOMATOM Force; Siemens Healthineers, Germany) following a standardized 3-phase imaging protocol. Phase 1 comprised a non-contrast scan from the lung apices to the heart base without contrast agent. Phase 2 involved left atrial CTA after intravenous injection of 85 mL iodinated contrast medium (Ultravist 370 mg I/mL; Bayer Schering Pharma) at a rate of 4-5 mL/s, followed by a saline flush. Scanning commenced once the contrast density in the ascending aorta reached 210 Hounsfield units (HU), effectively visualizing the left atrium and pulmonary veins. Phase 3, the delayed-phase imaging, was performed 5 minutes post-contrast injection from the tracheal carina to the heart base to assess late contrast enhancement and calculate the ECV (Figure 2).

**Figure 2:**
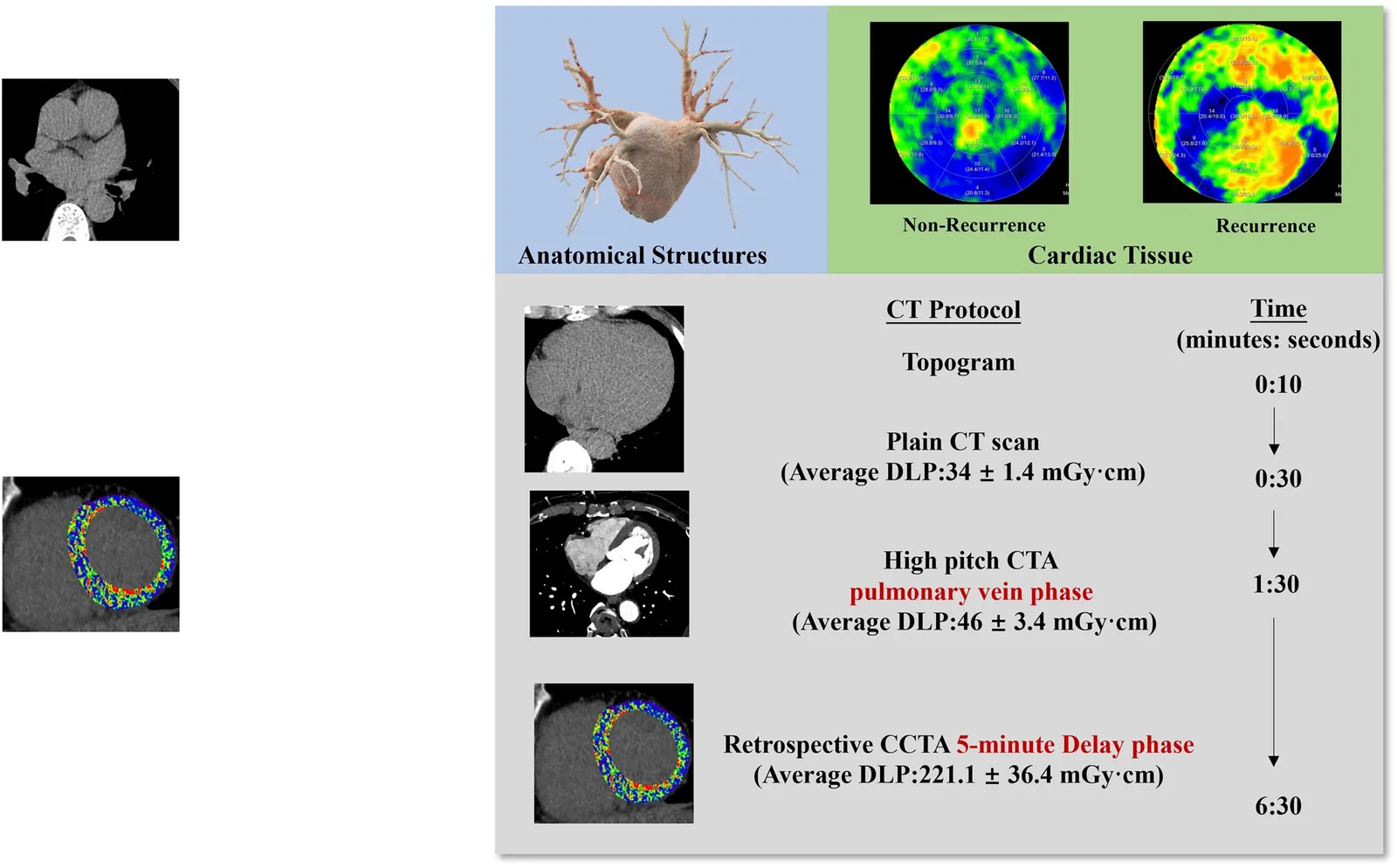
The flowchart of image acquisition methods.

Non-contrast (Phase 1) and Phase 2 acquisitions were performed using a high-pitch spiral mode with the following parameters: pitch, 1.9; detector collimation, 192 × 0.6 mm; gantry rotation time, 0.25 s; slice thickness, 1 mm; reconstruction increment, 0.7 mm. Images were reconstructed with an ADMIRE strength level of 3 and a Bv36 kernel. The tube voltage was set to 90 kV, and CARE Dose4D was applied for automatic tube current modulation.

For Phase 3, a retrospective ECG-gated acquisition was employed. A dual-energy protocol with tin filtration was used, with tube voltages of 80 and Sn150 kVp and a tube current of 387/215 mAs per rotation. Detector collimation was 192 × 0.6 mm, and the gantry rotation time was 0.25 s. The pitch was automatically adjusted between 0.15 and 0.31 according to the patient’s heart rate. Delayed phase images were reconstructed using an ADMIRE strength level of 2 and a Qr40 kernel, with a temporal resolution of 125 ms, a slice thickness of 1 mm, and an increment of 0.7 mm. The optimal cardiac phase was selected for reconstruction based on the heart rate.

### Myocardial ECV measurement

Hematocrit levels were measured within 24 hours of the CT scan to ensure accurate ECV calculation.^16^ ECV analysis was performed on a prototype software (Cardiac Functional Analysis, Syngo.via Frontier, Siemens Healthineers).^17^ Non-contrast and arterial phase images were subtracted (with a provided segmentation coefficient) and then registered with delayed phase images.^18^ Hematocrit values were input into the system, and myocardial ECV maps were automatically generated using the formula below.


(1) $$\mathrm{ECV}=\frac{{\Delta \mathrm{HU}}_{\mathrm{myocardium}}}{{\Delta \mathrm{HU}}_{\mathrm{blood}}}*(1-\mathrm{hematocrit}).$$

The ECV maps were overlaid onto the standard CTA images, allowing for the editing of myocardial contours. The results were then displayed in a 17-segment polar plot for detailed analysis.^19^

### Radiofrequency ablation procedure

All patients underwent RFA within 48 hours after the left atrial CTA. RFA was performed by experienced electrophysiologists who were blinded to the ECV results. The procedure involved standard pulmonary vein isolation, with additional linear lesions or substrate modification at the operator’s discretion based on intraoperative findings.^20^

### Follow-up protocol

Follow-up evaluations included clinical assessments at 1, 3, 6, 12, and 18 months; 12-lead electrocardiograms (ECGs) at each visit; 24-hour Holter monitoring at 1, 3, 6, 12, and 18 months; and additional monitoring if symptomatic episodes occurred. AF recurrence was defined as any atrial tachyarrhythmia lasting more than 30 seconds after a blanking period of 3 months post-ablation.

### Statistical analysis

Statistical analyses were conducted using R version 4.0.0 (R Foundation for Statistical Computing). Continuous variables are reported as mean ± standard deviation (SD) and compared using independent samples *t*-tests, 1-way analysis of variance, Wilcoxon signed-rank tests, or Kruskal-Wallis tests as appropriate. Categorical variables are presented as percentages. Youden’s index was used to determine the optimal cutoff value for dichotomizing ECV, dividing patients into high (>34.69%) and low (<34.69%) ECV groups. Cox proportional hazards regression was used for univariate analysis to identify potential predictors of AF recurrence, with variables having a *P* value <.05 included in multivariate analysis. Univariate Cox regression analysis was conducted to examine the correlation between the endpoint and baseline covariates. Variables were tested for collinearity using the variance inflation factor measure before inclusion in multivariable models. Variables with a *P* value <.10 in univariable analysis were included in the multivariable models, all of which demonstrated acceptably low multicollinearity. Survival curves were plotted using the Kaplan-Meier method and compared using the log-rank test. Three risk models were created to assess discriminative ability using the Concordance index (C-index): Model 1 included traditional clinical risk factors (age, heart failure, CHA_2_DS_2_-VASc, and HAS-BLED scores), Model 2 added left atrial (LA) volume index to Model 1, and Model 3 incorporated ECV into Model 2. The Net Reclassification Index (NRI), Integrated Discrimination Improvement (IDI), and Concordance index (C-index) were used to evaluate the clinical benefits and utility of traditional clinical AF prediction models versus combined ECV models.^21^^,^ ^22^  *P* value <.05 was considered statistically significant for all analyses.

## Results

### Patient characteristics

Of the 500 patients who underwent pre-ablation cardiac CT angiography, 101 were excluded due to previous AF ablation (*n = *13), prior myocardial infarction or coronary revascularization (*n = *22), contraindications to iodinated contrast media (*n = *16), loss to follow-up (*n = *27), or nondiagnostic CTA from respiratory motion artifacts (*n = *23), as detailed in Figure 1. The final analysis included 399 patients, with 91 experiencing AF recurrence and 308 without recurrence. The average follow-up period was 10.1 months (interquartile range, 8.1-13.6). Ninety-one (22.8%) experienced AF recurrence within 1-year post-ablation. Patients with recurrence were significantly older (65.12 ± 8.37 years vs. 61.18 ± 5.14 years; *P < .*01), heart failure (*P < .*01), and had higher CHA_2_DS_2_-VASc scores (*P < .*01). They exhibited lower hematocrit levels (0.40 ± 0.05 vs. 0.41 ± 0.02; *P < .*01), and larger left atrial volume index was greater in AF recurrence group (69.33 ± 7.85 mL/m² vs. 63.99 ± 8.34 mL/m²; *P < .*01). Baseline characteristics are summarized in Table 1.

**Table 1:** Baseline characteristics in non-paroxysmal atrial fibrillation patients with or without recurrence.

Characteristic	Total, (*n = *399)	No AF recurrence, (*n = *308)	AF recurrence, (*n = *91)	*P* value
Age (years)	62.08 ± 6.24	61.18 ± 5.14	65.12 ± 8.37	<.01
Male, *n* (%)	220 (55.42)	164 (53.25)	56 (62.92)	0.11
BMI, mean ± SD	20.29 ± 2.52	20.25 ± 2.48	20.43 ± 2.64	0.56
CHA_2_DS_2_-VASc (%)				<.01
1	82 (20.55)	40 (12.99)	42 (46.15)	
2	82 (20.55)	81 (26.30)	1 (1.10)	
3	97 (24.31)	86 (27.92)	11 (12.09)	
4	90 (22.56)	79 (25.65)	11 (12.09)	
>5	48 (12.03)	22 (7.14)	26 (28.57)	
HAS-BLED				<.01
0	59 (14.79)	32 (10.39)	27 (29.67)	
1	117 (29.32)	82 (26.62)	35 (38.46)	
2	121 (30.33)	103 (33.44)	18 (19.78)	
>3	102 (25.56)	91 (29.55)	11 (12.09)	
Heart failure (%)	117 (29.32)	47 (15.30)	70 (76.92)	<.01
Hypertension (%)	223 (55.89)	171 (55.52)	52 (57.14)	0.78
Diabetes (%)	170 (42.61)	135 (43.83)	35 (38.46)	0.36
Hypercholesterolemia (%)	158 (39.60)	136 (44.16)	22 (24.18)	<.01
Smoke (%)	156 (39.10)	126 (40.91)	30 (32.97)	0.17
HCT, mean ± SD	0.41 ± 0.03	0.41 ± 0.02	0.40 ± 0.05	<.01
Echocardiographic examination				
Left ventricular ejection fraction	0.96 ± 0.15	0.94 ± 0.10	1.00 ± 0.26	0.04
Left ventricular internal diameter at end-diastole (cm)	1.37 ± 0.12	1.37 ± 0.11	1.37 ± 0.16	0.77
Left ventricular internal diameter at end-systolic (cm)	0.63 ± 0.05	0.63 ± 0.04	0.63 ± 0.08	0.47
Interventricular septal thickness at end-diastole (cm)	5.23 ± 17.63	5.87 ± 20.03	3.04 ± 0.47	0.18
LV diameter at end-systolic (cm)	4.70 ± 0.32	4.72 ± 0.23	4.63 ± 0.53	0.13
Left atrium diameter (cm)	3.98 ± 0.44	3.95 ± 0.34	4.10 ± 0.66	0.03
Left atrium volume index (mL/m²)	65.21 ± 8.52	63.99 ± 8.34	69.33 ± 7.85	<.01
CT examination				
ECV (%), mean	33.72 ± 3.68	32.56 ± 2.05	37.64 ± 5.05	<.01

Except where indicated, data are means ± SDs. Categorical variables are shown with percentages in parentheses.

### Radiation dose

The average radiation dose for the non-enhanced CT was 1.4 ± 0.28 mSv (average dose-length product, 100.5 ± 6.7 mGy.cm). For delayed-phase ECV measurements, the average radiation dose was 3.1 ± 0.56 mSv (average dose-length product, 221.1 ± 36.4 mGy.cm).

### Myocardial extracellular volume fraction and segmental analysis

Patients with AF recurrence had significantly higher ECV values (37.6% ± 5.05%) compared to those without recurrence (32.6% ± 2.05%; *P < .*01) (Figure 3A). Analysis of the 17 left ventricular segments revealed that the basal inferior, basal anterolateral, and apical septal segments exhibited the most pronounced differences in ECV (all, *P < .*01). Figure S1 showing the ECV percentages in different segments of the left ventricle. ROC analysis determined an optimal ECV cutoff value of 34.69%, with an area under the curve (AUC) of 0.85 (95% CI: 0.79-0.90), sensitivity of 89%, specificity of 73%, positive predictive value of 92%, and negative predictive value of 66% (Figure 3B). Based on this established threshold, the baseline clinical and echocardiographic characteristics of the study population, stratified into high ECV (>34.69%) and low ECV (≤34.69%) groups, are summarized in Table S1. Kaplan-Meier survival analysis showed that patients with high ECV (>34.69%) had a significantly higher rate of AF recurrence (log-rank test; *P < .*01) (Figure 3C).

**Figure 3:**
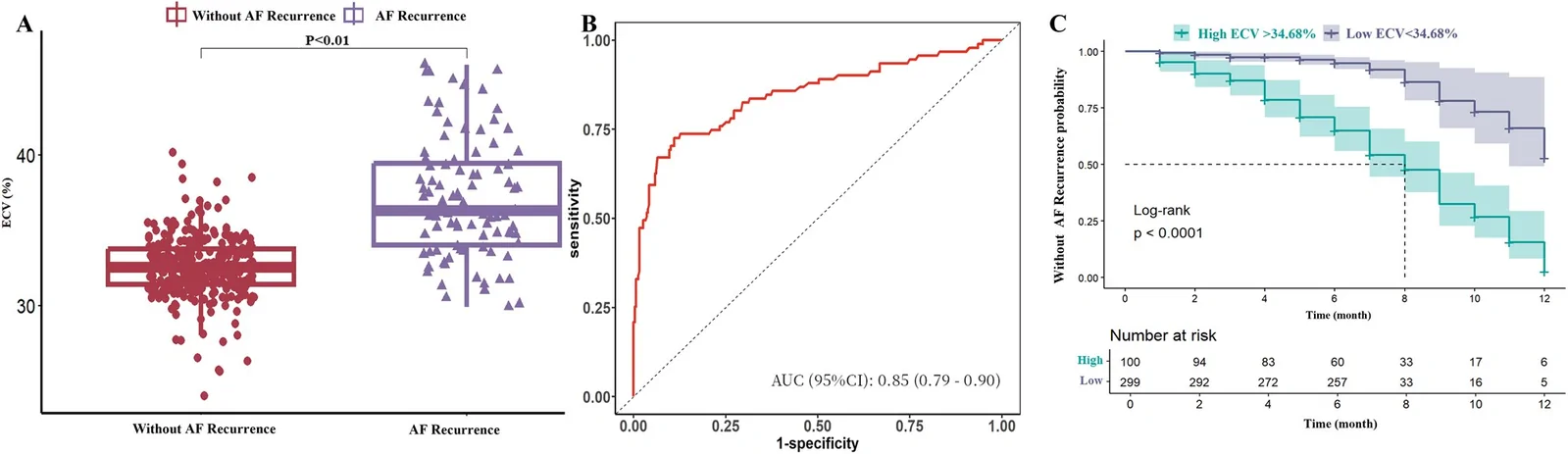
(A) The box plot comparing the ECV percentages between patients with and without AF recurrence. (B) A receiver operating characteristic (ROC) curve illustrating the diagnostic performance of ECV in predicting AF recurrence. (C) A Kaplan-Meier survival analysis plot comparing AF recurrence-free survival probability between high and low ECV groups.

### Cox regression analysis

Univariate Cox regression for time to AF recurrence identified age, CHA_2_DS_2_-VASc score, HAS-BLED score, hypercholesterolemia, heart failure, left ventricular internal diameter at end-systole, left ventricle ejection fraction, high ECV, and left atrial volume index as significant predictors. In the multivariate model, age (HR = 1.05; 95% CI: 1.03-1.09; *P < .*01), heart failure (HR = 1.27; 95% CI: 0.24-2.53; *P* < 0.01), LA volume index (HR = 1.03; 95% CI: 0.78-1.05; *P < .*01), left ventricle ejection fraction (HR = 1.43; 95% CI: 0.81-3.36; *P* = 0.01), and high ECV (HR = 1.84; 95% CI: 1.24-2.33; *P < .*01) remained independently associated with AF recurrence (Table 2).

**Table 2:** Univariate and multivariate Cox regression analysis for non-paroxysmal atrial fibrillation recurrence.

Parameter	Univariable analysis		Multivariable analysis	
	HR (95 CI%)	*P* value	HR (95 CI%)	*P* value
Age	1.06 (1.03-1.09)	<.01	1.05 (1.02-1.09)	<.01
Male sex	1.44 (0.93-2.21)	.10		
BMI	0.99 (0.91-1.07)	.78		
CHA_2_DS_2_-VASc	0.36 (0.18-0.71)	<.01	0.59 (0.27-1.28)	.18
HAS-BLED	2.37 (1.14-4.92)	<.01	0.98 (0.41-2.30)	.95
Heart failure	2.48 (1.26-5.31)	<.01	1.36 (0.33-2.42)	<.01
Hypertension	0.87 (0.57-1.33)	.52		
Diabetes	0.95 (0.62-1.45)	.80		
Hypercholesterolemia	0.54 (0.33-0.88)	.01	0.68 (0.42-1.12)	.13
Smoke	0.77 (0.50-1.20)	.25		
Left ventricular ejection fraction (%)	2.73 (1.51-4.57)	.03	1.43 (0.81-3.36)	.01
Left ventricular internal diameter at end-diastole (mm)	0.73 (0.39-1.37)	.33		
Left ventricular internal diameter at end-systolic (mm)	0.49 (0.30-0.81)	<.01	0.75 (0.53-1.05)	.09
Interventricular septal thickness at end-diastole (mm)	1.95 (0.96-3.98)	.07		
Left ventricle diameter at end-systolic (mm)	1.26 (0.27-5.96)	.77		
Left atrium diameter (mm)	1.55 (1.10-2.20)	.01	1.12 (0.76-1.67)	.57
Left atrium volume index(mL/m²)	1.08 (1.05-1.11)	<.01	1.10 (1.05-1.16)	<.01
ECV (%)	1.77 (1.54-2.02)	<.01	1.59 (1.36-1.85)	<.01

Data were processed with univariable and multivariate Cox regression analysis to predict recurrence 1 year after atrial fibrillation (AF) ablation. Data in parentheses are 95% CIs.

### Predictive value of ECV

Figure 4 illustrates 2 examples of the ECV in AF recurrence or without recurrence. Table 3 presents the C-index, NRI, and IDI for 3 models predicting AF recurrence of non-paroxysmal AF within 1 year. Model 1, which included traditional clinical risk factors (age, heart failure, CHA_2_DS_2_-VASc, and HAS-BLED scores), achieved a C-index of 0.61 (95% CI: 0.53-0.69). Enhancing this model by adding the LA volume and left ventricle ejection fraction index in Model 2 significantly improved the C-index to 0.71 (95% CI: 0.63-0.77), with an IDI of 0.29 (95% CI: 0.45-0.64) and an NRI of 0.26 (95% CI: 0.17-0.32). Further incorporating the ECV into Model 3 resulted in the highest predictive performance, with a C-index of 0.82 (95% CI: 0.74-0.86), an IDI of 0.66 (95% CI: 0.41-0.91), and an NRI of 0.55 (95% CI: 0.26-0.61).

**Figure 4:**
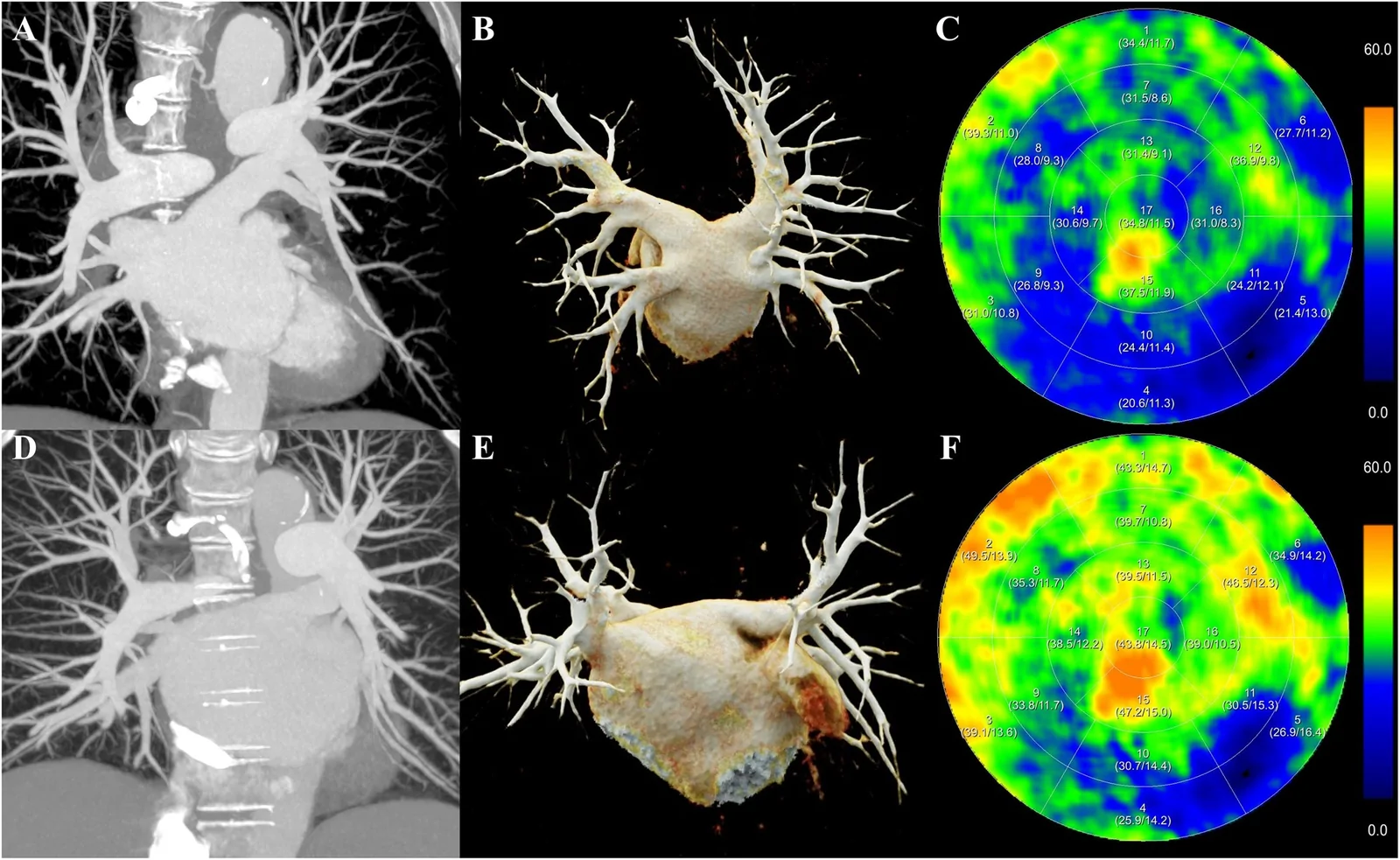
Two examples of ECV analysis obtained from delayed phase contrast-enhanced acquisition and AHA LV segmentation. (A-C) A 56-year-old female patient with a 1-year history of paroxysmal atrial fibrillation, exhibiting an average ECV of 29.9, experienced no recurrence within the subsequent year. (D and E) A 68-year-old male patient with a 4-year history of paroxysmal atrial fibrillation, presenting an average ECV of 40.4, demonstrated recurrence 6 months following radiofrequency ablation. A and D: Maximum-intensity-projection (MIP) coronal views from left atrial CTA; B and E: Volume Rendering Technique (VRT) 3-dimensional reconstructions of the left atrial. C and F: Color-coded bull’s-eye (17-segment) maps displaying segmental LV ECV values according to the American Heart Association model.

**Table 3:** C‐index, NRI, and IDI for predicting the recurrence of non-paroxysmal atrial fibrillation within 1 year by traditional risk models and the ECV combined model.

Model	C-index (95 CI%)	IDI (95 CI%)	NRI (95 CI%)
Model 1: Traditional clinical risk model	0.61 (0.53-0.69)	–	–
Model 2: Traditional clinical risk model + LA volume index + LVEF	0.71 (0.63-0.77)	0.29 (0.45-0.64)	0.26 (0.17-0.32)
Model 3: Traditional clinical risk model + LA volume index + LVEF + ECV	0.82 (0.74-0.86)	0.66 (0.41-0.91)	0.55 (0.26-0.61)

Model 1 traditional clinical risk included age, heart failure, CHA2DS2-VASc, and HAS-BLED; Model 2 included traditional clinical risk, LA volume index and LVEF; Model 3 included traditional clinical risk, LA volume index, LVEF, and ECV.

Abbreviations: C‐index, concordance index; IDI, integrated discrimination improvement; NRI, net reclassification index.

## Discussion

In this study, we demonstrated that CT-derived ECV of the myocardium is an independent predictor of AF recurrence after catheter ablation. In multivariable analysis adjusting for known clinical and imaging predictors, CT-derived ECV remained an independent predictor of AF recurrence. These findings suggest that preprocedural CT-derived ECV could serve as a noninvasive imaging biomarker for identifying patients at high risk of recurrence.

The fundamental biological mechanism linking left ventricular ECV to the recurrence of an atrial arrhythmia lies in the concept of a systemic, shared profibrotic substrate.^23^ Myocardial fibrosis is central to AF pathophysiology and recurrence, especially in non-paroxysmal AF where structural remodeling is more pronounced.^24^^,^ ^25^ Fibrosis results from excessive extracellular matrix accumulation, primarily driven by activation of profibrotic pathways such as transforming growth factor-β (TGF-β) signaling and the renin-angiotensin system.^26^^,^ ^27^ Clinical factors including aging, hypertension, and systemic inflammation trigger these pathways, leading to diffuse collagen deposition in both the atria and the ventricles simultaneously. Although atrial tissue is typically more prone to fibrosis, ventricular myocardium also remodels through these shared mechanisms.^28^^,^ ^29^ Therefore, elevated LV ECV is not merely a localized ventricular phenomenon, but rather a highly reproducible surrogate marker for the global profibrotic burden. This systemic fibrosis provides the structural substrate that facilitates arrhythmia maintenance and post-ablation recurrence. In addition, diffuse LV interstitial fibrosis is closely linked to impaired LV relaxation and elevated filling pressures, which can chronically increase left atrial pressure and volume load and thereby promote atrial stretch, remodeling, and electrical heterogeneity, pathophysiological conditions that reduce long-term ablation durability and favor recurrence. Prior CMR evidence has also shown that LV ECV expansion is associated with AF recurrence after catheter ablation, supporting the concept that LV ECV captures clinically relevant substrate beyond focal atrial scar.^30^

Importantly, our study focused exclusively on patients with non-paroxysmal (persistent or permanent) AF, a group known to have more pronounced atrial remodeling and myocardial fibrosis compared to those with paroxysmal AF.^31^ This study design likely enhanced the predictive value of fibrosis biomarkers such as ECV, since the greater and more heterogeneous fibrotic burden in non-paroxysmal AF may amplify the association between CT-derived ECV and the risk of recurrence. Previous cardiac MRI studies have confirmed that increased ECV correlates with histological fibrosis,^32^ and Tamura et al^33^ demonstrated that elevated ECV predicts adverse outcomes across multiple cardiac conditions. Consistent with these findings, our observation that patients with recurrence had higher CT-derived ECV further supports the role of diffuse myocardial fibrosis in increasing susceptibility to AF recurrence.

Regarding the biological state captured by pre-ablation ECV, it is important to distinguish chronic structural fibrosis from transient edema or acute inflammation. We acknowledge that ECV can be influenced by acute inflammatory processes. However, in our study, the optimal imaging timepoint was chosen during a clinically stable baseline prior to ablation. We strictly excluded patients with recent myocardial infarction or acute myocarditis. Furthermore, our protocol utilized a 5-minute delayed-phase acquisition, which approximates contrast equilibrium conditions and minimizes the influence of transient intravascular or acute edematous effects.^34^ Consequently, the elevated pre-ablation ECV in our cohort predominantly reflects established, chronic structural remodeling rather than acute, reversible inflammation, explaining its strong correlation with long-term AF recurrence.

While directly characterizing atrial tissue is theoretically ideal, it remains technically demanding. The thin atrial wall (typically 1-2 mm) is highly vulnerable to partial-volume effects and cardiac motion artifacts.^35^ In contrast, the thicker left ventricular myocardium allows for highly reliable and reproducible ECV quantification.^36^ Previous studies have confirmed the feasibility and accuracy of CT-derived ECV in various cardiac diseases,^37^ and robust correlations with MRI-ECV have been demonstrated, particularly in cardiac amyloidosis.^38^^,^ ^39^ Additionally, increased ECV has been associated with diastolic dysfunction and atrial remodeling and serves as a predictor of AF recurrence after pulmonary vein isolation in patients with hypertension.^40^ By focusing on patients with non-paroxysmal AF, who typically exhibit more extensive atrial remodeling and fibrosis, this study may enhance the predictive value of ECV compared to studies that included a broader spectrum of AF types.^41^ While contemporary CT with submillimeter spatial resolution makes regional atrial assessment technically feasible, we utilized global LV ECV as a robust, practical surrogate for the overall profibrotic burden. Future work incorporating atrial-specific approaches is warranted to determine their incremental value for procedural tailoring.

This study has some limitations. Foremost, and as the most important conceptual caveat of the present work, ECV was measured in the left ventricular myocardium rather than the left atrium, despite AF being primarily an atrial disease. Although our findings and previous literature suggest that LV ECV serves as a reliable surrogate for systemic profibrotic remodeling, it may not perfectly capture the localized severity or spatial heterogeneity of atrial fibrosis. We therefore explicitly underscore the LV-versus-LA ECV mismatch as the foremost limitation of this study. Future research should leverage advanced isotropic 3D datasets to include regional atrial ECV mapping. Direct atrial ECV quantification with optimized acquisition therefore remains a key priority for future prospective validation. Secondly, the single-center design and small sample size may limit the general applicability of our results; multi-center studies are necessary for validation. Thirdly, without serial imaging, we could not evaluate the temporal changes of ECV post-ablation or definitively completely exclude the minor contributions of subclinical edema. Finally, as there are no available multimodal data (such as electroanatomical mapping) in this retrospective cohort, direct spatial comparisons cannot be made.

In conclusion, myocardial ECV measured by cardiac CT emerges as a promising noninvasive biomarker for predicting AF recurrence after catheter ablation. These findings support existing theories on the role of atrial fibrosis in AF pathophysiology and highlight the potential of CTA-derived ECV as a practical tool for risk stratification.

## Supplementary Material

tqag149_Supplementary_Data

## Data Availability

The datasets used and/or analyzed during the current study are available from the corresponding author on reasonable request.
