# Supplementary material for: Pre-ablation dual-energy CT-derived extracellular volume fraction predicts recurrence after radiofrequency ablation in non-paroxysmal atrial fibrillation
Source: Br J Radiol. 2026 Jun 18;99(1187):2277–85. doi: 10.1093/bjr/tqag149 (PMC13687765; doi:10.1093/bjr/tqag149)
Supplement: Supplementary file 1 [file tqag149_supplementary_data.zip › supply.docx]

**Supplementary Materials**

**Pre-ablation Dual-Energy CT-derived Extracellular Volume Fraction Predicts Recurrence After Radiofrequency Ablation in Non-paroxysmal Atrial Fibrillation.**

Table S1. Baseline Clinical and Echocardiographic Characteristics of Patients Stratified by ECV.

| Characteristic | Total  (n = 399) | Low ECV  (n = 299) | High ECV  (n = 100) | *P* |
| --- | --- | --- | --- | --- |
|  |  |  |  |  |
| Age (y) | 62.08 ± 6.24 | 61.76 ± 5.05 | 63.02 ± 8.87 | 0.18 |
| Male, *n* (%) | 221 (55.53) | 164 (54.85) | 57 (57.58) | 0.64 |
| BMI, Mean ± SD | 20.29 ± 2.52 | 20.21 ± 2.52 | 20.52 ± 2.49 | 0.28 |
| CHA_2_DS_2_-VASc (%) |  |  |  | <.01 |
| 1 | 82 (20.55) | 78 (26.09) | 4 (4.00) |  |
| 2 | 97 (24.31) | 81 (27.09) | 16 (16.00) |  |
| 3 | 90 (22.56) | 78 (26.09) | 12 (12.00) |  |
| 4 | 48 (12.03) | 22 (7.36) | 26 (26.00) |  |
| >5 | 82 (20.55) | 40 (13.38) | 42 (42.00) |  |
| HAS-BLED |  |  |  | <.01 |
| 0 | 59 (14.79) | 27 (9.03) | 32 (32.00) |  |
| 1 | 117 (29.32) | 83 (27.76) | 34 (34.00) |  |
| 2 | 121 (30.33) | 102 (34.11) | 19 (19.00) |  |
| >3 | 102 (25.56) | 87 (29.10) | 15 (15.00) |  |
| Heart failure (%) | 91 (22.81) | 25 (8.36) | 66 (66.00) | <.01 |
| Hypertension (%) | 223 (55.89) | 168 (56.19) | 55 (55.00) | 0.84 |
| Diabetes (%) | 170 (42.61) | 136 (45.48) | 34 (34.00) | 0.04 |
| Hypercholesterolemia (%) | 158 (39.60) | 135 (45.15) | 23 (23.00) | <.01 |
| Smoke (%) | 156 (39.10) | 123 (41.14) | 33 (33.00) | 0.15 |
| HCT, Mean ± SD | 0.41 ± 0.03 | 0.42 ± 0.02 | 0.39 ± 0.05 | <.01 |
| Echocardiographic examination | |  |  |  |
| Left ventricle ejection Fraction | 0.63 ± 0.05 | 0.63 ± 0.04 | 0.63 ± 0.08 | 0.84 |
| Left ventricle internal diameter at end-diastole (cm) | 5.23 ± 1.76 | 5.77 ± 1.08 | 3.61 ± 1.69 | 0.29 |
| Left ventricle internal diameter at end- systolic (cm) | 0.96 ± 0.15 | 0.94 ± 0.09 | 0.99 ± 0.26 | 0.11 |
| Interventricular Septal Thickness at end-diastole (cm) | 4.70 ± 0.32 | 4.72 ± 0.25 | 4.64 ± 0.48 | 0.11 |
| LV diameter at end-systolic (cm) | 1.37 ± 0.12 | 1.37 ± 0.11 | 1.36 ± 0.14 | 0.43 |
| Left atrium diameter (cm) | 3.98 ± 0.44 | 3.95 ± 0.31 | 4.08 ± 0.68 | 0.06 |
| Left atrium volume index (mL/m²) | 65.21 ± 8.52 | 64.49 ± 8.21 | 67.35 ± 9.09 | <.01 |

*Abbreviations:* ECV = extracellular volume fraction; BMI = body mass index; HCT = hematocrit; SD = standard deviation. Low ECV was defined as an ECV ≤ 34.69%, and High ECV was defined as an ECV > 34.69% based on the ROC-derived optimal cutoff

Fig S1 The bar chart showing the ECV percentages in different segments of the left ventricle, segmented according to the American Heart Association (AHA) model between patients with and without AF recurrence.
